# Supplementary material for: Recurrence of post-term pregnancy and associated factors among women who delivered at Kilimanjaro Christian Medical Centre in northern Tanzania: A retrospective cohort study
Source: PLoS One. 2023 Apr 4;18(4):e0282078. doi: 10.1371/journal.pone.0282078 (PMC10072474; doi:10.1371/journal.pone.0282078)
Supplement: Attachment — Submitted filename: PONE-D-21-33495_reviewer_dm.pdf [file pone.0282078.s001.pdf]

PLOS ONE

Recurrence of post-term pregnancy and associated factors among women who delivered at Kilimanjaro Christian Medical University Centre in Northern Tanzania: A retrospective cohort study

--Manuscript Draft--

**Manuscript Number:**	PONE-D-21-33495
**Article Type:**	Research Article
**Full Title:**	Recurrence of post-term pregnancy and associated factors among women who delivered at Kilimanjaro Christian Medical University Centre in Northern Tanzania: A retrospective cohort study
**Short Title:**	Recurrence of post-term pregnancy and associated factors among women in Northern Tanzania
**Corresponding Author:**	Modesta Paschal Mitao, Medical doctor<br>Kilimanjaro Christian Medical College: Kilimanjaro Christian Medical University College<br>Kilimanjaro, TANZANIA, UNITED REPUBLIC OF
**Keywords:**	Recurrence; post-term pregnancy; adverse pregnancy outcomes; birth registry; Tanzania
**Abstract:**	Background: Post-term pregnancy is a health problem of clinical importance and; tends to recur in subsequent pregnancies. Post-term pregnancy increases the risk of maternal, perinatal and fetal morbidity, and mortality. This study provided evidence for informed interventions to reduce the recurrence risk of post-term pregnancy towards reducing associated maternal and fetal complications. The study aimed to determine the recurrence risk of post-term pregnancy and associated factors among women delivered at KCMC referral hospital.<br>Methodology: This retrospective cohort study used KCMC zonal referral hospital medical birth registry cohort data for 43472 women delivered between 2000 and 2018. Data were analyzed using STATA version 15 software. Log-binomial regression with robust variance estimator determined the factors associated recurrence of post-term pregnancy adjusted for other factors.<br>Results: A total of 43472 women were analyzed. The proportion of post-term pregnancy was 11.4%, and the recurrence risk was 14.8%. The recurrence risk of post-term pregnancy was increased when a woman had a history of previous post-term pregnancy (aRR: 1.75; 95%CI: 1.44, 2.11). Advanced maternal age, i.e., ≥35years (aRR: 0.80; 95%CI: 0.65, 0.99), having secondary and higher education (aRR: 0.8; 95%CI: 0.66, 0.97), and being employed (aRR : 0.68; 95%CI: 0.55, 0.84) decreased the recurrence risk of post-term pregnancy. Women with recurrence of post-term pregnancy had a higher risk of delivering newborns weighed ≥4000gm (aRR: 5.05; 95%CI: 2.80, 9.09).<br>Conclusion: Post-term pregnancy is associated with recurrence risk in subsequent pregnancies. Clinical counselling of women at risk of post-term pregnancy and timely management is recommended to prevent adverse neonatal and maternal outcomes.
**Order of Authors:**	Modesta Paschal Mitao, Master of epidemiology and applied biostatistics
	Winfrida Mwita, Master of epidemiology and applied biostatistics
	Cecilia Antony, Master of epidemiology and applied biostatistics
	Hamidu Adinan, Master of epidemiology and applied biostatistics
	Benjamin Shayo, Master of medicine in obstetrics and gynaecology
	Caroline Amour, Master of epidemiology and applied biostatistics
	Innocent B Mboya, Master of epidemiology and applied biostatistics
	Michael Johnson Mahande, Master of epidemiology and applied biostatistics
**Additional Information:**	
**Question**	**Response**

Powered by Editorial Manager® and ProduXion Manager® from Aries Systems Corporation

**Financial Disclosure**<br><br>Enter a financial disclosure statement that describes the sources of funding for the work included in this submission. Review the submission guidelines for detailed requirements. View published research articles from *PLOS ONE* for specific examples.<br><br>This statement is required for submission and **will appear in the published article** if the submission is accepted. Please make sure it is accurate.<br><br>**Unfunded studies**<br>Enter: *The author(s) received no specific funding for this work.*<br><br>**Funded studies**<br>Enter a statement with the following details:<br>• Initials of the authors who received each award<br>• Grant numbers awarded to each author<br>• The full name of each funder<br>• URL of each funder website<br>• Did the sponsors or funders play any role in the study design, data collection and analysis, decision to publish, or preparation of the manuscript?<br>• **NO** - Include this sentence at the end of your statement: *The funders had no role in study design, data collection and analysis, decision to publish, or preparation of the manuscript.*<br>• **YES** - Specify the role(s) played.<br><br>* typeset	The author(s) received no specific funding for this work.
**Competing Interests**<br><br>Use the instructions below to enter a competing interest statement for this submission. On behalf of all authors, disclose any competing interests that could be perceived to bias this work—acknowledging all financial support and any other relevant financial or non-financial competing interests.<br><br>This statement is required for submission	The authors have declared that no competing interests exist.

and **will appear in the published article** if the submission is accepted. Please make sure it is accurate and that any funding sources listed in your Funding Information later in the submission form are also declared in your Financial Disclosure statement.<br><br>View published research articles from *PLOS ONE* for specific examples.<br><br>**NO authors have competing interests**<br><br>Enter: *The authors have declared that no competing interests exist.*<br><br>**Authors with competing interests**<br><br>Enter competing interest details beginning with this statement:<br><br>*I have read the journal's policy and the authors of this manuscript have the following competing interests: [insert competing interests here]*<br><br>* typeset	
**Ethics Statement**<br><br>Enter an ethics statement for this submission. This statement is required if the study involved:<br><br>• Human participants<br>• Human specimens or tissue<br>• Vertebrate animals or cephalopods<br>• Vertebrate embryos or tissues<br>• Field research<br><br>Write "N/A" if the submission does not require an ethics statement.<br><br>General guidance is provided below. Consult the submission guidelines for detailed instructions. **Make sure that all information entered here is included in the Methods section of the manuscript.**	Ethical clearance for the KCMC Medical birth registry was granted from the ethics committee at KCMC and the National Ethics of Norway. Also, similar clearance has been granted from the Tanzanian Ministry of health, Commission for Science and Technology. Verbal consent was sought from each mother prior to the interview, which was conducted just after the woman had given birth. Participation was voluntary and had no impact on the management women would receive. Mothers were free to refuse to reply to any single question. For privacy and confidentiality, unique identification numbers were used to both identify and then link mothers with child records.<br>Ethical approval to carry out the current study was obtained from the Kilimanjaro Christian Medical College Research Ethics and Review Committee (KCMU-CREC) with clearance number PG 03/2020.

Powered by Editorial Manager® and ProduXion Manager® from Aries Systems Corporation

**Format for specific study types**<br><br>**Human Subject Research (involving human participants and/or tissue)**<br>• Give the name of the institutional review board or ethics committee that approved the study<br>• Include the approval number and/or a statement indicating approval of this research<br>• Indicate the form of consent obtained (written/oral) or the reason that consent was not obtained (e.g. the data were analyzed anonymously)<br><br>**Animal Research (involving vertebrate animals, embryos or tissues)**<br>• Provide the name of the Institutional Animal Care and Use Committee (IACUC) or other relevant ethics board that reviewed the study protocol, and indicate whether they approved this research or granted a formal waiver of ethical approval<br>• Include an approval number if one was obtained<br>• If the study involved *non-human primates*, add *additional details* about animal welfare and steps taken to ameliorate suffering<br>• If anesthesia, euthanasia, or any kind of animal sacrifice is part of the study, include briefly which substances and/or methods were applied<br><br>**Field Research**<br><br>Include the following details if this study involves the collection of plant, animal, or other materials from a natural setting:<br>• Field permit number<br>• Name of the institution or relevant body that granted permission	
**Data Availability**<br><br>Authors are required to make all data underlying the findings described fully available, without restriction, and from the time of publication. PLOS allows rare exceptions to address legal and ethical concerns. See the PLOS Data Policy and FAQ for detailed information.	Yes - all data are fully available without restriction

Powered by Editorial Manager® and ProduXion Manager® from Aries Systems Corporation

A Data Availability Statement describing where the data can be found is required at submission. Your answers to this question constitute the Data Availability Statement and **will be published in the article**, if accepted.<br><br>**Important:** Stating 'data available on request from the author' is not sufficient. If your data are only available upon request, select 'No' for the first question and explain your exceptional situation in the text box.<br><br>Do the authors confirm that all data underlying the findings described in their manuscript are fully available without restriction?	
**Describe where the data may be found in full sentences. If you are copying our sample text, replace any instances of XXX with the appropriate details.**<br><br>• If the data are **held or will be held in a public repository**, include URLs, accession numbers or DOIs. If this information will only be available after acceptance, indicate this by ticking the box below. For example: *All XXX files are available from the XXX database (accession number(s) XXX, XXX.).*<br>• If the data are all contained **within the manuscript and/or Supporting Information files**, enter the following: *All relevant data are within the manuscript and its Supporting Information files.*<br>• If neither of these applies but you are able to provide **details of access elsewhere**, with or without limitations, please do so. For example:<br><br>*Data cannot be shared publicly because of [XXX]. Data are available from the XXX Institutional Data Access / Ethics Committee (contact via XXX) for researchers who meet the criteria for access to confidential data.*<br><br>*The data underlying the results presented in the study are available from (include the name of the third party*	Yes-all data are fully available without restriction

and contact information or URL).<br>• This text is appropriate if the data are owned by a third party and authors do not have permission to share the data.<br><br>* typeset	
Additional data availability information:	

Manuscript
Click here to access/download;Manuscript;Manuscript for submission.docx

Recurrence of post-term pregnancy and associated factors among women who delivered at Kilimanjaro Christian Medical Centre in northern Tanzania: A retrospective cohort study

Modesta Mitao[1]*, Winfrida Mwita[1], Cecilia Antony[1], Hamidu Adinan[1], , Benjamin Shayo[3], Caroline Amour[1,2], Innocent B.Mboya[1,2,4], Michael Johnson Mahande[1,2,3]

[1]Institute of Public Health, Department of Epidemiology and Biostatistics, Kilimanjaro Christian Medical University College (KCMUCo), P. O. Box 2240, Moshi Tanzania

[2]Institute of Public Health, Department of Community Medicine, Kilimanjaro Christian Medical University College (KCMUCo), P. O. Box 2240, Moshi, Tanzania

[3]Department of Obstetrics and Gynaecology, Kilimanjaro Christian Medical Centre, P.O BOX 3010, Moshi, Tanzania

[4]School of Mathematics, Statistics & Computer Science, University of KwaZulu Natal, Pietermaritzburg, Private Bag X01, Scottsville 3209, South Africa

***Corresponding author:** Modesta Mitao, KCMUCo, P. O. Box 2240, Moshi Tanzania, 12

E-mail:modestamitao@gmail.com

Author E-mails:

MM: modestamitao@gmail.com

WM: winnie_mgole@yahoo.com

CA:cecyantony2013@gmail.com

HA:h.amdu@yahoo.com

BS: benjamin.shayo@gmail.com

CA: lyneamour@gmail.com

IBM: ib.mboya@gmail.com

MJM: jmmahande@gmail.com

Abstract

Background: Post-term pregnancy is a health problem of clinical importance and; tends to recur in subsequent pregnancies. Post-term pregnancy increases the risk of maternal, perinatal and fetal morbidity, and mortality. This study provided evidence for informed interventions to reduce the recurrence risk of post-term pregnancy towards reducing associated maternal and fetal complications. The study aimed to determine the recurrence risk of post-term pregnancy and associated factors among women delivered at KCMC referral hospital.

Methodology: This retrospective cohort study used KCMC zonal referral hospital medical birth registry cohort data for 43472 women delivered between 2000 and 2018. Data were analyzed using STATA version 15 software. Log-binomial regression with robust variance estimator determined the factors associated recurrence of post-term pregnancy adjusted for other factors.

Results: A total of 43472 women were analyzed. The proportion of post-term pregnancy was 11.4%, and the recurrence risk was 14.8%. The recurrence risk of post-term pregnancy was increased when a woman had a history of previous post-term pregnancy (aRR: 1.75; 95%CI: 1.44, 2.11). Advanced maternal age, i.e., ≥35years (aRR: 0.80; 95%CI: 0.65, 0.99), having secondary and higher education (aRR: 0.8; 95%CI: 0.66, 0.97), and being employed (aRR: 0.68; 95%CI: 0.55, 0.84) decreased the recurrence risk of post-term pregnancy. Women with recurrence of post-term pregnancy had a higher risk of delivering newborns weighed ≥4000gm (aRR: 5.05; 95%CI: 2.80, 9.09).

Conclusion: Post-term pregnancy is associated with recurrence risk in subsequent pregnancies. Clinical counselling of women at risk of post-term pregnancy and timely management is recommended to prevent adverse neonatal and maternal outcomes.

Keywords: Recurrence, post-term pregnancy, adverse pregnancy outcomes, birth registry, Tanzania.

Introduction

Post-term pregnancy extends beyond 42 weeks of gestation (294 days) after the first day of the last menstrual period [1]. Post-term pregnancy is a health problem of clinical importance with recurrence risk in subsequent pregnancies. It is associated with fetal and neonatal morbidity and mortality. The global incidence of post-term pregnancy varies by country, the review of randomized clinical trials done in industrialized, low, and middle-income countries reported incidence of between 3% and 14% [2]. In Europe, a study done in 13 countries reported the incidence of post-term pregnancies ranges between 0.5% and 10% [3,4].

Studies in sub-Saharan Africa focused on the prevalence of post-term pregnancy. For example, a study in Kenya reported a prevalence of 10% [5], while a study in Ethiopia reported a prevalence of 6% [6].

Previous cohort studies from high-income countries have reported recurrence of post-term pregnancy in subsequent pregnancies ranging from 15% in the Netherland to 16.9% in the USA [7,8]. There is a paucity of data on the recurrence rate of post-term pregnancies in sub-Saharan Africa. This study will give the magnitude of the problem for evidence-based interventions by clinicians, pregnant women and policymakers.

Post-term pregnancy is influenced by maternal age, height, male fetal gender, paternal genetics, and behavioral characteristics [8]. On the other hand, factors associated with the recurrence of post-term pregnancy include high BMI (>35 kg/m^2), white race, familiar factor, not graduating from high school [7,9,10]. Evidence from inter-generational recurrence studies has demonstrated that mothers born post-term have an increased risk of having post-term pregnancy. Similarly, post-term fathers are more likely to trigger post-term pregnancy in their partners [9].

Post-term pregnancy is a health problem associated with an increased risk of maternal, perinatal, fetal, and neonatal morbidity and mortality [3]. Post-term mothers often undergo cesarean delivery and are at increased risk of experiencing postpartum hemorrhage [11–14]. Cesarean section is associated with increased infections, injury to nearby organs, increased need for blood transfusion, death and high associated costs [14].

These women are at increased risk of experiencing shoulder dystocia, which can cause maternal trauma to the bladder, anal sphincter, rectum and arm fracture to the newborn [15].

Intervention such as labor induction at term and beyond term demonstrated a reduction in adverse infant and maternal outcomes such as cesarean section [16]. Also, interventions such as skilled birth attendance coverage, availability of basic and comprehensive emergency obstetric care, promotion of health facility delivery are implemented by the Tanzania Ministry of health to improve maternal and neonatal outcomes including post-term pregnancy [17].

In a systematic review done in Europe, USA, and France reported risk of perinatal mortality in post-term pregnancy is around 5.8% [4]. Post-term pregnancies recur in subsequent pregnancies, but their magnitude and associated factors are unknown in Tanzania. The study aimed to determine the recurrence risk of post-term pregnancy and associated factors among women delivered at KCMC zonal referral hospital in northern Tanzania. This study will help uncover the knowledge gaps and generate data to help design interventions to improve maternal and newborn health and save maternal and children's lives.

Methods

Study design and setting

This is retrospective cohort study utilized birth cohort data from the Kilimanjaro Christian Medical Center (KCMC) zonal referral hospital in Moshi Municipality, Northern Tanzania. The KCMC medical birth registry was established as a pilot in 1999 in collaboration with the Medical Birth Registry of Norway and the University of Bergen.

Data were collected from the department of obstetrics and gynecology as well as the pediatric departments at KCMC hospital. KCMC is one of the four recognized referral hospitals in Tanzania. It serves patients from the Kilimanjaro region (main catchment area), nearby regions, including Arusha, Tanga, Manyara, and other regions of Tanzania. Pregnant women with complications are referred to KCMC for management, while women in the local community may come to deliver by self-referral. The KCMC medical registry has recorded an average of 4,000 births per year. The main socio-economic activities of the Kilimanjaro region include agriculture, tourism and industrial activities [18–20].

Study population, sample size, and sampling

The parent study included all singleton pregnant women who provided informed consent and recorded in the KCMC medical birth registry between 2000 and 2018. The study excluded participants with missing information on either date of deliveries or date of last menstrual period. There were 62920 deliveries recorded in the KCMC medical birth registry between 200-2018. 62 deliveries with missing hospital numbers were excluded as it was used to link mother's information with their newborns. Also, the following deliveries were excluded 5674 deliveries with gestational age before 28 weeks and after 44 weeks, 3250 multiple births as they are likely to be preterm than post-term from literature, and 2273 deliveries with the missing date of last menstrual period because these are the variables used to compute

gestational age which was used to categorize recurrence of post-term pregnancy, the outcome of interest. There were 51490 deliveries from 43742 mothers. We excluded 43472 deliveries without subsequent deliveries in data analysis for recurrence risk of post-term and associated factors.; hence 8025 deliveries with subsequent deliveries were analyzed (Figure **1**).

Variables

The main outcome variables in this study were recurrence of post-term pregnancy. Post-term pregnancy is defined as a pregnancy extending beyond 42 weeks of gestation (294 days) from the date of the last menstrual period [1]. This variable was generated by calculating the difference between the date of the child's birth and the first day of a woman's last menstrual period. Post-term pregnancy was recorded as a binary variable: no post-term pregnancy "<42 weeks of gestational" and post-term pregnancy "≥42 weeks of gestational". Recurrence of post-term pregnancy was defined as the occurrence of one or more post-term pregnancies in a subsequent pregnancy.

Independent variables included maternal socio-demographic characteristics and obstetric characteristics. The study's selection and categorization of independent variables were based on previous literature [18–20].

Maternal socio-demographic characteristics

Mothers' background characteristics include Mother's age (≤19, 20-34, and ≥35 years), occupation (formal employment, i.e., professions, services and informal employment, i.e., housewife, farmer, students and business), education levels (no formal/primary education and secondary/higher education level), and area of residence (rural and urban). In addition, the study also included maternal body mass index (BMI) (underweight (<18.5 kg/m^2), normal weight (18.5-24.9 kg/m^2) and overweight/obesity (≥ 25 kg/m^2).

Obstetric characteristic

Obstetric characteristics include birthweight which is defined as a newborn's weight at birth (low birthweight <2500gm, normal weight 2500gm- 3999gm, and overweight ≥4000gm) [21]; Child status (Liveborn, Transferred to NICU and Perinatal death which is defined as deaths occurring in the first week of life and stillbirths [22]), ANC visit (<4 and ≥ 4 visits).

Data processing and analysis

Data analysis was performed using STATA version 15 statistical software (Stata Corp, College Station, TX). Descriptive statistics were summarized using the frequency and percentage for categorical variables, and mean with standard deviations was used for numeric variables. The Chi-square test was used to compare the difference in proportions post-term pregnancy by participant characteristics.

The multivariable Log-binomial regression with robust estimator was used to determine the factors associated with recurrence of post-term pregnancy.

Independent variables with a p-value of <5% were entered in the multivariable regression model one after another to estimate its contribution on recurrence of post-term pregnancy , the outcome of interest. A variable was a confounder if its inclusion in the model changed the relative risk by 10%. Also, multicollinearity between exposures were checked using pairwise correlation. A p-value of <5% was considered statistically significant.

Results

Socio-demographic and obstetric characteristics of women in a subsequent pregnancy

A total of 8025 deliveries were analyzed. The recurrence risk of post-term pregnancy in subsequent pregnancies (two or more pregnancies) was 14.8% (143/965). The proportion of post-term pregnancy varies across socio-demographic and obstetrics characteristics in the subsequent pregnancies (**Table 1**). 10.4% of women who had no formal and primary education had a recurrence of post-term pregnancy compared to 7.5% of those who had secondary and higher education; P-value <0.001. 10% of unemployed women had recurrence of post-term pregnancy compared to 6.1% of employed women; P-value<0.001. 13.5% of women who gave birth to children weighing 4000gm or more had recurrence of post-term pregnancy; P-value<0.001.

Factors associated with recurrence of post-term pregnancy

The factors associated with the recurrence of post-term pregnancy are displayed in Table **2**. In crude analysis, previous post-term pregnancy (cRR: 1.80; 95%CI: 1.52, 2.14) was significantly associated with an increased risk of recurrent postterm pregnancy in the subsequent pregnancies. Other factors such as young maternal age (15-19) years (cRR: 1.21; 95%CI: 0.70, 2.08), 20-24 years (cRR: 1.15; 95%CI: 0.95, 1.39) increased women's likelihood of recurrence post-term pregnancy but these were not statistically significant. On the other hand, secondary and higher education (cRR: 0.72; 95%CI: 0.63-0.83) and employed women (cRR: 0.61; 95% CI: 0.51, 0.74) were significantly associated with a lower risk of recurrent post-term pregnancy compared to counterparts.

In adjusted analysis, the previous history of post-term pregnancy remained significantly associated with an increased risk of post-term pregnancy (aRR: 1.75; 95%CI: 1.44, 2.11). Advanced maternal age, i.e., ≥35years (aRR: 0.80; 95%CI: 0.65, 0.99), and employed women (aRR: 0.68; 95%CI: 0.55, 0.84) had lower risk of recurrence of post-term pregnancy compared to their counterparts. Women who had recurrent post-term pregnancies had a 5-fold higher risk of delivering heavier newborns (aRR: 5.05; 95%CI: 2.80, 9.09).

Discussion

This study aimed to determine the recurrence risk of post-term pregnancy and associated factors among women who delivered at KCMC from 2000 to 2018. The proportion of women who had post-term pregnancies during their first recorded pregnancy was 11.4%. The recurrence risk of post-term pregnancy was 14.8%. In addition, a previous history of post-term pregnancy was associated with an increased risk of post-term pregnancy. In contrast, advanced maternal age, being employed were significantly associated with a lower risk of recurrence of

post-term pregnancy. Furthermore, recurrent post-term pregnancy increased women's likelihood of delivering heavier newborns.

The recurrence risk of post-term pregnancy in the study setting was 14.8%. This figure is approximate similar to 15% which was previously reported in the Netherland [8]. But it is lower compared to 16.9% and 19.9% which were reported in the USA and Denmark [7,23]..Difference in management guidelines of post-term pregnancy across countries may also explain variations in the recurrence risk, in countries which have adopted WHO recommendation of induction of labor at 41 weeks would have low rate of recurrence of post-term pregnancy [16].

In this study, advanced maternal age (>35 years) was associated with lower risk of recurrent post-term pregnancy than women aged 25-34 years. Similarly, women with secondary and higher education had a lower risk of recurrence of post-term pregnancy. This is consistent with the previous study in the USA [7]. It is possible that higher education influences awareness of health education. Therefore, educated women were more likely to seek health care by attending health facilities early when the due date had passed when compared with their counterparts with lower education levels [24].

The previous history of previous post-term pregnancy was associated with an increased risk of recurrent post-term pregnancy. Our finding is in-congruent with previous studies in the Netherlands, Denmark, Scotland, and the USA [7,8,23,25]. This could be because post-term pregnancy is influenced by genetic factors responsible for controlling parturition, which can persist between successive pregnancies [7].

The study strength is that it is the first in Tanzania to evaluate the recurrence risk and factors associated with post-term pregnancy. Therefore, the study estimated the burden of recurrent post-term pregnancy and associated factors in northern Tanzania. The KCMC Medical birth

registry data used in this study has large sample size, hence enough statistical power that increase precision of the estimates. In addition, the cohort design nature using maternally linked data to create reproductive history for each woman enabled estimating the recurrence risk of post-term pregnancy and its determinants.

Despite these strengths, it is worth noting that this study utilized secondary data, which might have compromised the data quality and thereby the estimates. This includes missing values in outcome and exposures. Some reproductive parameters to study recurrent post-term depend on maternal recall memories, subject to recall bias. For example, mothers were asked to recall the date of their last normal menstrual period at the time. Since the majority of the women can't exactly remember the date of their last menstrual period, this can lead to over or underestimating gestational age and consequently post-term pregnancy. Also, if this happened, it could lead to differential misclassification bias. We acknowledge the missing of ultrasound scans at the first trimester, which is most accurate means of estimating gestation age.

In addition, since our study utilized data from the referral setting, there is a possibility of selection bias. However, more than two-thirds of our study participants were self-referred.

Conclusion

The proportion of post-term pregnancies in this study was 11.4%, while the recurrence risk of post-term pregnancy 14.8%. Recurrence of post-term pregnancy was associated with having history of previous post-term pregnancy, advanced maternal age ≥35years, being employed, and delivering newborns weighed ≥4000gm.

Recommendations

The study recommends health workers to provide health education to women on risk factors associated with recurrence of post-term pregnancy such history of post-term pregnancy.

Hence, women at risk should attend to health facility early for proper and timely management for best pregnancy outcomes.

Women should be encouraged by health workers to attend at health facility when the due date has been reached without initiation of labour for interventions such as labour induction to prevent adverse perinatal and maternal outcomes.

Abbreviation

ACOG	American College of Obstetricians and Gynecologist
ANC	Antenatal Care
FIGO	Federation of international of Gynecologists and Obstetricians
KCMC	Kilimanjaro Christian Medical Centre
KCMUCo	Kilimanjaro Christian Medical University Collage
MoHCDGEC	Ministry of Health, Community Development, Gender, Elderly and Children
WHO	World Health Organization

Ethical consideration

Ethical clearance for the KCMC Medical birth registry was granted from the ethics committee at KCMC and the National Ethics of Norway. Also, similar clearance has been granted from the Tanzanian Ministry of health, Commission for Science and Technology. Verbal consent was sought from each mother prior to the interview, which was conducted just after the woman had given birth. Participation was voluntary and had no impact on the management women would receive. Mothers were free to refuse to reply to any single question. For privacy and confidentiality, unique identification numbers were used to both identify and then link mothers with child records.

Ethical approval to carry out the current study was obtained from the Kilimanjaro Christian Medical College Research Ethics and Review Committee (KCMU-CREC) with clearance number PG 03/2020.

Consent for publication

Not applicable

Availability of data and materials

Data will be made available

Competing Interest

None of the authors has any conflict of interest in the content of this manuscript.

Acknowledgement

We acknowledge all nurses and doctors at KCMC who took part in data collection and all women who consented to provide their information making this study possible. Also, we would like to acknowledge Dr. Rafiki Mjema, Dr. Joachim Frank Magoma and Dr. Joel Msafiri Francis who reviewed this work and for their valuable suggestion.

Author's contributions

MM, IM,CA, MJM contributed to the design of the study. MM performed the statistical analysis and drafted the manuscript. WM, CA and HA participated in statistical analysis and drafting of the manuscript. BS, CA,IBM, and MJM edited the final draft of the manuscript and approved the final manuscript.

REFERENCE

1. ACOG (American College of Obstetricians and Gynecologists). Management of Postterm Pregnancy. ACOG Practice bulletin no. 55. Obstet Gynecol. 2004;104:639-4

2. Middleton P, Shepherd E, Crowther CA. Induction of labour for improving birth outcomes for women at or beyond term. Cochrane Database Syst Rev. 2018;2018(5):26–8.

3. Galal M, Symonds I, Murray H, Petragilia F, Smith R. Postterm pregnancy. FVV OBGYN. 2012;4(3):175–87.

4. Vayssière C, Haumonte JB, Chantry A, Coatleven F, Debord MP, Gomez C, et al. Prolonged and post-term pregnancies: Guidelines for clinical practice from the French College of Gynecologists and Obstetricians (CNGOF). Vol. 169, European Journal of Obstetrics and Gynecology and Reproductive Biology. Elsevier Ireland Ltd; 2013. p. 10–6.

5. Kavoo-Linge, Rogo KO. Factors influencing early perinatal mortality in a rural district hospital. East Afr Med J [Internet]. 1992 Apr [cited 2019 Dec 8];69(4):181–7. Available from: http://www.ncbi.nlm.nih.gov/pubmed/1644026

6. Mengesha HG, Lerebo WT, Kidanemariam A, Gebrezgiabher G, Berhane Y. Pre-term and post-term births: predictors and implications on neonatal mortality in Northern Ethiopia. BMC Nurs [Internet]. 2016 Dec 5 [cited 2019 Dec 9];15(1):48. Available from: http://bmcnurs.biomedcentral.com/articles/10.1186/s12912-016-0170-6

7. Kistka ZAF, Palomar L, Boslaugh SE, DeBaun MR, DeFranco EA, Muglia LJ. Risk for postterm delivery after previous postterm delivery. Am J Obstet Gynecol. 2007;196(3):241.e1-241.e6.

8. Kortekaas JC, Kazemier BM, Ravelli ACJ, De Boer K, Van Dillen J, Mol BW, et al. Recurrence rate and outcome of postterm pregnancy, a national cohort study. Eur J Obstet Gynecol Reprod Biol. 2015 Oct 1;193:70–4.

9. Morken NH, Melve KK, Skjaerven R. Recurrence of prolonged and post-term gestational age across generations: Maternal and paternal contribution. BJOG An Int J Obstet Gynaecol. 2011;118(13):1630–5.

10. Heslehurst N, Vieira R, Hayes L, Crowe L, Jones D, Robalino S, et al. Maternal body mass index and post-term birth: a systematic review and meta-analysis. Vol. 18, Obesity Reviews. Blackwell Publishing Ltd; 2017. p. 293–308.

11. Caughey AB, Stotland NE, Washington AE, Escobar GJ. Maternal and obstetric complications of pregnancy are associated with increasing gestational age at term. Am J Obstet Gynecol [Internet]. 2007 Feb 1 [cited 2019 Dec 9];196(2):155.e1-155.e6. Available from: https://www.sciencedirect.com/science/article/abs/pii/S0002937806011781

12. De Los Santos-Garate AM, Villa-Guillen M, Villanueva-García D, Vallejos-Ruíz ML, Murguía-Peniche MT. Perinatal morbidity and mortality in late-term and post-term pregnancy. NEOSANO perinatal network's experience in Mexico. J Perinatol. 2011 Dec;31(12):789–93.

13. McCoy J, Downes KL, Srinivas SK, Levine LD. Postdates induction with an unfavorable cervix and risk of cesarean*. J Matern Neonatal Med. 2018 Mar 22;1–5.

14. Tarimo CS, Mahande MJ, Obure J. Prevalence and risk factors for caesarean delivery following labor induction at a tertiary hospital in North Tanzania: A retrospective cohort study (2000-2015). BMC Pregnancy Childbirth. 2020;20(1):1–8.

15. Sandström A, Cnattingius S, Wikström AK, Stephansson O. Labour dystocia-risk of recurrence and instrumental delivery in following labour-a population-based cohort study. BJOG An Int J Obstet Gynaecol. 2012;119(13):1648–56.

16. World Health Organization. WHO recommendations: Induction of labour at or beyond term. WHO recommendations: Induction of labour at or beyond term. 2018. 39 p.

17. Ministry of Health. The National Road Map Strategic Plan to Improve Reproductive, Maternal, Newborn, Child and Adolescent Health in Tanzania - One Plan II. 2016;(June):142.

18. Mmbaga, Blandina Theophil; Lie Rolv Terje;Olomi, Raimos; Mahande, Michael Johnson; Kvåle, Gunnar ; and Daltveit AK. BMC Pediatrics Full text Cause-specific neonatal mortality in a neonatal care unit in Northern Tanzania a registry based cohort study. BMC Pediatr. 2012;12:116.

19. Mboya IB, Mahande MJ, Obure J, Mwambi HG. Predictors of perinatal death in the presence of missing data: A birth registry-based study in northern Tanzania. PLoS One [Internet]. 2020;15(4):1–22. Available from: http://dx.doi.org/10.1371/journal.pone.0231636

20. Mwanamsangu AH, Mahande MJ, Mazuguni FS, Bishanga DR, Mazuguni N, Msuya SE, et al. Maternal obesity and intrapartum obstetric complications among pregnant women: Retrospective cohort analysis from medical birth registry in Northern Tanzania. Obes Sci Pract. 2020;6(2):171–80.

21. UNICEF-WHO.Low birthweight estimates: Levels and trends 2000–2015. Geneva World Heal Organ. 2019;4(3):3–9.

22. World Health Organization (WHO). Perinatal and neonatal mortality Country.British

Medical Journal.2006;281(6254): 1567

23. Olesen AW, Basso O, Olsen J. Risk of recurrence of prolonged pregnancy. Br Med J. 2003 Mar 1;326(7387):476.

24. Dickson KS, Darteh EKM, Kumi-Kyereme A, Ahinkorah BO. Determinants of choice of skilled antenatal care service providers in Ghana: analysis of demographic and health survey. Matern Heal Neonatol Perinatol. 2018;4(1):1–8.

25. Ashton G, Bhattacharya S, Shetty A. Repeat induction of labour for post-term pregnancy. J Obstet Gynaecol (Lahore) [Internet]. 2018;38(5):724. Available from: https://doi.org/10.1080/01443615.2018.1444393

 Click here to access/download;Figure;Figure and tables.docx

Table 1 : Socio-demographic and obstetric characteristics of women delivered at KCMC in subsequent pregnancy, 2000-2018 (N=8025)

Characteristics	Total (n)	Post-term	P-value
**Previous post-term**			
No	7060	580 (8.2)	<0.001
Yes	965	143 (14.8)	
**Mother's Age (years)[a]**			
15-19	111	14(12.6)	0.056
20-24	1112	116(10.4)	
25-34	5075	458 (9.0))	
≥35	1706	133 (7.8)	
**Residence [b]**			
Rural	2573	238 (9.3)	0.615
Urban	5446	485 (8.9)	
**Mother's education level [c]**			
No formal and primary education	4087	426 (10.4)	<0.001
Secondary and Higher	3930	294 (7.5)	
**Mother's occupation [d]**			
Unemployed	5963	596 (10.0)	<0.001
Employed	2037	125 (6.1)	
**BMI[e]**			
Underweight	466	36 (7.7)	0.394
Normal weight	3543	321 (9.1)	
Overweight/obesity	2183	211 (9.7)	
**ANC visit [f]**			
<4	2680	209 (7.8)	0.006
≥4	5273	510 (9.7)	
**Child characteristics**			
**Child status [g]**			
Liveborn	6833	620 (9.1)	0.172
Transferred to NICU	995	92 (9.2)	
Perinatal death	163	78 (4.29)	
**Birth weight(g) [h]**			
<2500	640	22 (3.4)	<0.001

2500-3999	6926	642 (9.3)
≥4000	437	59 (13.5)

a: n=8004; b: n=8019; c: n=7971; d: n=8000; e: n=6192; f: n=7992; g: n=8003; h: n=7953
*****Frequencies (n) do not tally to the total due to missing values in these variables

Table 2 : Factors associated with recurrence of post-term pregnancy among women delivered at KCMC in subsequent pregnancy, 2000-2018 (N=8025)

Characteristics	cRR (95%CI)	P-value	aRR (95%CI)	P-value
**Previous post-term**				
No	1.00		1.00	
Yes	1.74 (1.52-2.14)	<0.001	1.74 (1.44-2.11)	<0.001
**Mother's Age(years)**				
25-34	1.00		1.00	
15-19	1.21(0.70-2.08)	0.491	1.37(0.74-2.53)	0.311
20-24	1.15 (0.95-1.39)	0.153	1.20(0.96-1.49)	0.102
≥35	0.86 (0.71-1.03)	0.101	0.80 (0.65-0.99)	0.036
**Mother's education level**				
No formal and Primary education	1.00		1.00	
Secondary and Higher	0.72 (0.63-0.83)	<0.001	0.80 (0.66-0.97)	0.020
**Mother's occupation**				
Unemployed	1.00		1.00	
Employed	0.61 (0.51-0.74)	<0.001	0.72 (0.56-0.91)	<0.001
**ANC visit**				
<4	1.00		1.00	
≥4	1.26(1.08-1.48)	0.003	1.27 (1.07-1.52)	0.007
**BMI**				
Normal weight	1.00		1.00	
Underweight	0.85 (0.61-1.19)	0.345	1.17 (0.61-1.19)	0.348
Overweight/Obese	1.07 (0.90-1.26)	0.443	1.27 (0.92-1.29)	0.319
**Child status**				
Live	1.00		1.00	
Transferred to NICU	1.01 (0.84-1.27)	0.752	1.04 (0.80-1.36)	0.759
Perinatal death	0.60(0.3-1.13)	0.114	1.11 (0.45-1.64)	0.747
**Birth weight(g)**				
<2500	1.00		1.00	
2500-3999	2.70 (1.78-4.09)	<0.001	3.09 (1.66-4.04)	<0.001
≥4000	4.02 (2.51-6.44)	<0.001	5.05 (2.80-9.09)	<0.001

***** cRR- Crude relative risk

* aRR-Adjusted relative risk

*Adjusted for previous post-term, mother's age, education, occupation, ANC visit, BMI, child status, and child's birthweight.

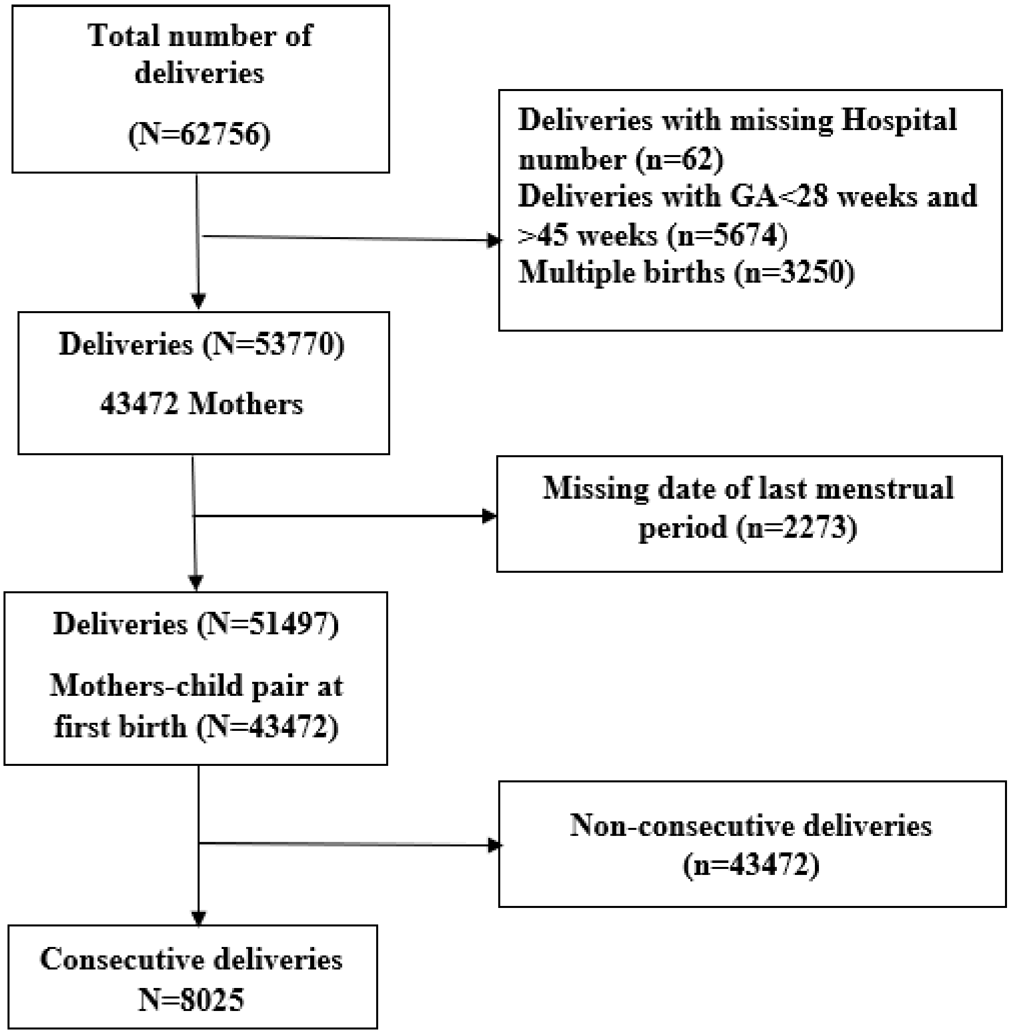

Figure 1 : Flowchart of the number of deliveries from 2000 to 2018 recorded in the KCMC medical birth registry.

Click here to access/download
Supporting Information - Compressed/ZIP File Archive
New Compressed (zipped) Folder.zip
