# Supplementary material for: Recurrence of post-term pregnancy and associated factors among women who delivered at Kilimanjaro Christian Medical Centre in northern Tanzania: A retrospective cohort study
Source: PLoS One. 2023 Apr 4;18(4):e0282078. doi: 10.1371/journal.pone.0282078 (PMC10072474; doi:10.1371/journal.pone.0282078)
Supplement: Attachment — Submitted filename: PONE-D-21-33495_Comments_dm.docx [file pone.0282078.s002.docx]

**Comments:**

Abstract:

Data analysis: I am wondering why to use robust variance estimator in log-binomial model if the model is converged. Often multivariable log-binomial model fails to converge and, in that case, we use Poisson model with robust variance estimator. Please clarify what exactly was done.

Line 94: Please write as “This is a retrospective study….”

Line 111: Please write “2000-2018”

Line 113: Please check with the figure-1. Here it is mentioned after 44 weeks whereas in the figure it is mentioned >45 weeks, which are not same.

Line 150/151: Please see the comments in the abstract.

Line 152: Please justify using P value of 5% in bivariate analysis for considering for multivariable model.

Final comment: The manuscript can be published after addressing the comments.
