# Supplementary material for: Recurrence of post-term pregnancy and associated factors among women who delivered at Kilimanjaro Christian Medical Centre in northern Tanzania: A retrospective cohort study
Source: PLoS One. 2023 Apr 4;18(4):e0282078. doi: 10.1371/journal.pone.0282078 (PMC10072474; doi:10.1371/journal.pone.0282078)
Supplement: Attachment — Submitted filename: Response to Reviewers.docx [file pone.0282078.s003.docx]

PONE-D-21-33495

Recurrence of post-term pregnancy and associated factors among women who delivered at Kilimanjaro Christian Medical University Centre in Northern Tanzania: A retrospective cohort study

|  | **Examiners comments** | **Authors responses** |
| --- | --- | --- |
|  | **Introduction** |  |
| 1. | The authors should consider revising the abstract. In the background section, the sentence looks like a conclusion (P 2, L 28-30). As mentioned in the results, 'the risk of 14.8%' should be a proportion. In the end, the conclusion is not based on study results and therefore needs revision | The abstract in background section has been revised, the concluding sentence have been removed (P2, L28-29). The conclusion section has been revised to base on study results. |
| 2. | Firstly introduction section needs to be revised as structured an introduction with sharp objectives Rationale of analyzing this data without considering time (2 decades old data). | The introduction section has been structured to include burden, from global to country level (P3, L52-65), risk factors (P3, L66-70), outcomes (P4, L71-77), interventions in place (P4, L86-88), problem statement, and justification of the study (P4, L89-94)  The objectives are now smart (P4, L95-97). |
| 3. | Introduction section should be more organized. For example, the effect of post-term birth has been mentioned in several paragraphs. It is not clear how the present study will help clinicians and policymakers (P 3, L 64,65)? | The introduction section has been revised to show the effect of post-term pregnancy (P4, L76-82). Proper explanation has been given to show how present study will help clinicians and policymakers (P3, L68-70). |
| 4. | There is a lack of texts to indicate the weaknesses of the previous studies. Furthermore, how the present study will contribute to our understanding of the problem. Why is there a lack of studies in low-income countries? | Texts to indicate the weaknesses of previous studies was added (P4, L86-89).  The explanation on how the present study will contribute to our understanding of the problem was given (P4, L90-91).  There is a lack of studies in low-income countries because scholars have not explored this area. Recommendation have been done to emphasize studies to be done. |
| 5. | Please revise the sentence (P 3, L 69-71). The last sentence is not meaning any sense. The study's rationale should be modified to convince the study's usefulness. | The sentence (P3, L 69-71) has been revised. The last sentence has been removed.  The study rationale has been revised to convince the study usefulness (P4, L94-97). |
|  | **Methodology** |  |
| 6. | Why and how data were merged to a converge model instead of considering poison model. | The Multivariable Log-binomial regression with robust estimator was used to determine the factors associated with recurrence of post-term pregnancy, it was used because it take account of binomial data and repeated events, and poison model take account of count data. |
| 7. | Is the manuscript technically sound, and do the data support the conclusions? | The manuscript has technically sound piece of scientific research, this is because the study has large power due to use of large sample size (8025 participants). Also, recurrence risk has been estimated to determine a burden of recurrence of post-term pregnancy and a relative risk to determine associated factors. |
| 8. | Has the statistical analysis been performed appropriately and rigorously? | Yes, the statistical analysis has been performed appropriately by taking into account recurrence of events. |
| 9. | Is the manuscript presented in an intelligible fashion and written in Standard English? | The manuscript has now been reviewed to be in an intelligible and standard English. |
| 10. | Line 94: Please write as “This is a retrospective study….” | The study design has been revised as suggested (Line 101). |
| 11. | Line 111:Please write “2000-2018” | The study was rewrite “2000-2018”(Line 112) |
| 12. | Line 113: Please check with the figure-1. Here it is mentioned after 44 weeks whereas in the figure it is mentioned >45 weeks, which are not same. | The gestational weeks has been revised to >44 weeks to present post-term pregnancy in the figure 1. |
| 13. | The data collected covered the time of almost two decades. A significant change in social and service utilization may occur during that period. The authors did not consider the time in the analysis. There is also a change in cesarean section (CS) rates, and the history of CS may influence the post-term rate in subsequent pregnancies | The analysis has been revised to take account of the two decades. We have adjusted for decades as shown in table 2.  There were no significant difference in recurrence of post-term pregnancy between the two decades. |
| 14. | Line 152: Please justify using P value of 5% in bivariate analysis for considering for multivariable model. | The consideration of variables to be entered in Multivariable model has been changed from using the one with P-value <5% to all important variables according to literature were entered in final models. |
| 15. | The methods section should describe how each variable was measured. Please add texts to explain how women's LMP, height, and weight were collected? | The method section has been revised to include how each variable was measured. To include how women’s LMP (P8, L140-141), height, and weight were collected (P8, L155). |
| 16. | How many women are with their first and second births available? It may be interesting to see the recurrence risk in the second birth. | There were 43472 mothers with their first birth and 8025 women with second births as shown in **fig 1**.  The recurrence risk of post-term pregnancy was calculated among mothers who had second births. |
| 17. | How the missing information was dealt with in the analysis. For example, table 2 shows a significant number of missing values. The authors should clarify how many women were included in the adjusted analysis. | The observations with the missing data on variable used to calculate outcome were deleted. In the analysis, there were no significant differences in post-term births and perinatal death whether you consider missing values or not.  The number of women included in adjusted analysis has been shown on a **table 2** heading (N=8025). |
|  | **Results** |  |
| 18. | The authors should describe the distribution of preterm, term, and post-term births by year to get general ideas about the burden of those events in the hospital | The distribution of preterm, term, and post-term births by year was done using line graph in **fig 2**. |
|  | **Discussion** |  |
| 19.. | Discussion section: This section needs more work to make the article interesting. The essential findings are not highlighted adequately. It is implied that the induction of labor or delivery by cesarean section may influence the results. | The discussion section has been revised where essential findings are highlighted adequately, with an inclusion of implications of induction of a labour and cesarean section (P11-12, L230-234) |
|  | What was the recall period of LMP date information? | The recall period of LMP date depended on when the mother booked for her first ANC visit. |
|  | It is also not convincing how education may influence the occurrence of post-term births. | It is possible that higher education influences awareness of health education. Therefore, educated women were more likely to seek health care by attending health facilities early when the due date had passed when compared with their counterparts with lower education levels (P11, L220-223). |
